# Religiosity predicts negative attitudes towards science and lower levels of science literacy

**DOI:** 10.1371/journal.pone.0207125

**Published:** 2018-11-27

**Authors:** Jonathon McPhetres, Miron Zuckerman

**Affiliations:** Department of Clinical and Social Sciences in Psychology, University of Rochester, Rochester, NY, United States of America; Coventry University, UNITED KINGDOM

## Abstract

Past research suggests that religion and science may conflict on which is a better tool for explaining the world. This conflict implies that religiosity might negatively impact both attitudes toward science and science knowledge. However, past research has focused mostly on religious affiliation and has not consistently identified such a relation using a general religiosity measure that assesses religious beliefs and religious practice. Using two large, nationally representative datasets as well as two original datasets, and controlling for relevant demographic variables, four studies (*N =* 9,205) showed that general measures of religiosity are negatively associated with science knowledge, a relation that was partially mediated by an association between religiosity and negative attitudes toward science. Study 2 also showed that parents’ reports about their religiosity and its role in their children’s upbringing predicted, some 20 years later, their children’s attitudes toward science. The studies are correlational but the longitudinal relations in Study 2 suggests that religiosity might undermine science literacy.

## Introduction

With several key scientific topics (e.g., climate change, safety of GMOs) [1] dividing the American public, and recent reports placing the United States at 27^th^ out of 64 countries in science performance [2], public support and trust in science are more important than ever. However, attitudes toward science and science literacy are usually viewed as an educational issue, a perspective that does not consider the role of religion as a potential detractor from the value of science. Although surveys show that the general public, and even scientists, may think that science and religion do not conflict [3, 4], some evidence to the contrary exists, both in research [5] and in reviews of the literature [6, 7]. Given the relative cultural importance of religion in the United States [8], and increasing skepticism towards science [7], this issue merits an empirical investigation. In the present studies, we used two large national datasets and two additional datasets collected by the authors to examine the relations between religiosity and both attitudes towards science and science literacy.

The potential conflicts between science and religion can be viewed as limited or general. The limited version is that the conflict exists only for a few topics where science contradicts religious assertions, such as the origin of the Earth and the origin of humans [5,9]. Additionally, some scientific research—such as stem cell research—may have moral and ethical implications to which religious people object [5, 10, 11]. Outside of these specific epistemological and moral contentions, according to the limited view, we would not expect religious teachings or believers to oppose science.

However, a number of research findings suggest that the conflict between science and religion is more general, at least within the US. For example, greater religiosity is related to less favorable views toward scientific innovations and nanotechnology [11, 12], and religious people are less likely to choose careers in science [13, 14]. Survey data also shows that religious beliefs are negatively correlated with scientific consensus on a number of issues (e.g., vaccinations [15], climate change [16],) even when such issues do not directly challenge religious claims. While some researchers posit that objections towards nanotechnology and vaccinations may be driven by concerns about morality and scientists “playing God” [17, 18], the fact remains that these topics do not conflict with religious teachings per se—that is, we are not aware of religious texts which speak directly about nanotechnology, vaccinations, or climate change. Thus, religious people justify their opposition to some scientific concepts in terms of moral and religious beliefs in the same way that holders of a particular political ideology will oppose an idea in terms of economic or social justification. In contrast, Christian religious texts *do* discuss the creation of the world (versus evolution) and the sanctity of life (versus stem cell research). Viewed in this light, the conflict appears to address a more general epistemological dispute about whether science or religion is a better tool for understanding and explaining the world [19]. Thus, the general conflict hypothesis implies that religious people have more negative attitudes and possibly less trust towards science as a source of information. In the present studies, we tested whether this general conflict results in more negative attitudes towards science and in lower levels of scientific literacy.

Three previous studies examined the relation between religion and scientific literacy, using data from the General Social Survey (GSS) in 2006 [5, 9] or in 2008 [10]. Two of the studies [5, 10] found no such relationship. The third study [9] found that religious fundamentalists and Catholics (but not mainline Protestants, Jews, and Muslims) possessed less science knowledge than those participants who stated they were not affiliated with any religious group.

Some of the differences among these studies may be due to how science knowledge and religiosity were defined. For example, two of the studies [5, 9] included apparently “contested” items (questions about the Big Bang and continental drift) where a wrong answer may reflect a religious conviction rather than a lack of knowledge. The studies also differed in how religiosity was measured, such that the comparison between religious and non-religious people focused on religious affiliation and fundamentalism but did not involve the same groups.

Religiosity is defined differently by many researchers. However, Allport’s [20] construct of intrinsic religiosity, which refers to belief and identity, has been described as “…the *sine qua non* theory about what it means to be truly and appropriately religious” ([21], pg. 1). Indeed, widely used measures focus on religious beliefs, practice, and identity (e.g., [22, 23]), as those concepts are applicable to many different religious groups [23, 24]. Yet, the three studies just discussed [5, 9, 10] focused on specific combinations of religious affiliation and fundamentalism when investigating science attitudes and knowledge, which lends to a rather limited interpretation of the relation between religion and science. True, some religious groups do endorse teaching which conflict more directly with science (for example, literalist teachings about the creation of humans and the earth), but that is not the subject matter that we address. Instead, we aimed to investigate whether elements of religiosity which are common to most religions uniquely contribute to science rejection, not whether specific religious groups know more or less about science than others. Further, members of the same religious group can differ in the extent to which they endorse certain teachings, so a focus on affiliation ignores valuable individual differences. Finally, including affiliation and belief in an equation simultaneously creates problems of multicollinearity and makes interpretation of each variable difficult. For example, we assume that most people who identify themselves as Christian will score higher on a belief measure than people who identify themselves as atheists. For a minority, however, this difference will be reversed, raising the question of understanding where they are in terms of religiosity. In addition, any analysis of this minority will be hampered by low statistical power. We, therefore, proceed to examine only aspects of religiosity—religious beliefs and religious practice—which are common to most, if not all, religious groups [21–24].

Finally, whereas past research has found that the relation between religiosity and science depends on how the religiosity variable is measured or categorized [5, 9, 10], in the present research we demonstrate that this relation is robust and generalizable to broad, yet conceptually similar, measures of religiosity. Our present operationalization of religiosity also departs from past research due to differences between disciplines. Previous research on the relation between religiosity and science knowledge has so far come from sociologists; social psychologists have paid little attention to this area of research. Whereas social psychologists are interested in the individual differences in religious beliefs, sociologists may be more interested in how the religious traditions themselves influences attitudes towards science which results in more of a focus on religious affiliation. In our investigation, we sought to take a different approach simply to provide a different perspective on the relation between religion and science.

It’s also important to note that the studies reviewed above consider the relation between religion and science from a US perspective. While the above patterns may be stronger in the US—or may even only be true for the US—it is currently unknown whether these relations are true for other countries around the world. In evaluating the past and current research, these limitations should be kept in mind.

### The present studies

Taken as a whole, the relation between religiosity and science knowledge has not been empirically established; at the most, it has been examined in relation to specific religious affiliations and fundamentalism. We sought to address some limitations of the previous investigations. First, to increase statistical power, we used GSS data from all five separate surveys that included science knowledge questions (2006–2016). Second, in addition to the GSS [25] data (examined in Study 1), we also tested the relation between religiosity and science knowledge in another large, independently collected dataset (Study 2), the Longitudinal Study of American Youth (LSAY [26]) and two more original datasets (Study 3–4).

Third, assuming that affiliation and fundamentalism are rather limited measures of religiosity, we used instead general religiosity scales that included a number of questions about both religious faith and practice. Thus, one important contribution of the present research is to operationalize religiosity differently from past research. Here we examined whether general measures of religiosity (focusing only on belief and practice) consistently predict negative science attitudes and lower levels of science literacy.

Fourth, we examine the “limited” versus “general” conflict hypotheses by using two measures of science knowledge. First, in Studies 1 and 2, we present zero-order correlations between religiosity and a measure of science knowledge which includes questions about “contested” topics such as evolution—we expected religiosity to be negatively related to this measure. However, if the conflict between science and religion were more general and not limited to specific topics, we would expect the negative relation to persist after removing the contested items from the science knowledge scale. Therefore, following Johnson et al. [10], we used a science knowledge measure that excluded “contested” topics (e.g., evolution, the Big Bang, and continental drift), thus testing the general conflict hypothesis.

Fifth, in Study 2, we examined whether the relation between participants’ religiosity and attitudes towards science begins with the religiosity of their parents. A large research literature indicates that parents exert strong religious influence on their offspring’s’ beliefs and behavior in both childhood and adolescence [27–31] as well as in adulthood [32–34] (but see Hunsberger and Brown [35], who reported that parents’ religious attendance and whether the children received religious education was related to their children’s attendance when the children were in the high teens but not when they were in their early 30s). One problem with this evidence, however, is that in most cases it was based on children’s reports of their own and of their parents’ religiosity. Nevertheless, the findings suggest a possible path from parents’ religiosity to children’s religiosity. The present study examined whether this link exists, using measures of parents’ religiosity that were obtained from the parents, and measures of children’s religiosity and science knowledge that were obtained from the children, separately, over 20 years later.

Finally, we also tested whether the predicted negative relation between religiosity and science knowledge is mediated by attitudes towards science. The rationale for such mediation is straightforward. To the extent that religious people view science as invalid, irrelevant, or morally suspect, they will be less interested in learning science, both formally and informally.

Of course, this logic might also imply that another possible mediator is the amount of learning that a person has done. Education is linked to science knowledge because much of this knowledge is learned in schools. Religiosity is linked to education because it appears that those with lesser with lesser religious beliefs are more likely to seek out higher levels of education [36]. This logic suggests that the relation between science knowledge and religiosity will be mediated by education. On the other hand, religiosity may not necessarily translate into an overall anti-education bias; Catholic schools, for example, actually excel on the usual criterion of standardized tests [37]. Therefore, it is possible that the mediator is not general formal education, typically measured by the number of years spent on schooling, but the type of education one chooses to get. Indeed, we noted above that religious people are less likely to choose careers in science [13, 14]. Since the data available to us concerned *length* rather than *nature* of participants’ education, we were not sure whether we would find support for mediation.

We should note that our rationale implies a directional connection from higher religiosity to lesser science knowledge. We obviously could not obtain experimental evidence to test this prediction. However, whereas Study 1 is purely correlational, Study 2 is in part longitudinal, which is the preferable design to test directional hypotheses when experimental evidence cannot be collected.

## Study 1

### Method

#### Participants

The National Opinion Research Center at the University of Chicago collects the GSS from a random sample of the English-speaking population in the US. We used the data from five different cohorts participating in the survey in 2006–2016. After deleting respondents with missing data on at least one variable, we examined responses of 4,627 participants (2,535 females). The participants had an average age of 47.78 (*SD* = 16.54) and an average education of 14.09 years (*SD* = 2.92). A majority of the participants (81%) were white, 10% were black, and 9% identified as other. A list of all the demographics that the analyses controlled for is presented in the Results section.

#### Measuring religiosity

We identified five items that measured religious beliefs and behaviors: confidence in the existence of God, how often respondent attends religious services, how often the respondent prays, how religious the respondent is, and strength of religious affiliation (questions about fundamentalism and the frequency of nonreligious activities at church were excluded). Beliefs about God were measured on a scale ranging from 1 (*don’t believe*) to 6 (*know God exists without doubts*); frequency of attendance was indicated on a scale from 0 (*never*) to 8 (*more than once a week*); frequency of prayer was indicated on a scale from 1 (*never*) to 6 (*several times a day*). The remaining questions were answered on a scale from 1 (*not at all*) to 4 (*very much/very strong*). The five items were standardized and combined into a religiosity composite (α = .87).

#### Measuring scientific knowledge

We used the seven-item scientific knowledge measure that was previously used by Johnson et al., [10]. Participants received one point for each correct response and zero points for incorrect or “Don’t know” responses. As participants answered question 7b (how long it takes for the earth to go around the sun) only if they correctly answered question 7a (whether the earth goes around the sun), they were given two points if they answered both questions correctly. As such, scores on the scale had a 0–8 range with higher scores indicating higher scientific knowledge. The questions are displayed in the upper half of Table 1. However, we also report correlations for the total science knowledge measure, which includes 3 additional “contested” questions about evolution, the big bang, and continental drift.

**Table 1:** Science knowledge questions used in studies 1 and 2. Total correct responses were summed; 7b was displayed only if 7a was answered correctly.

**Study 1: GSS**
1. It is the father’s gene that decides whether the baby is a boy or a girl. 2. Electrons are smaller than atoms. 3. The center of the earth is very hot. 4. Lasers work by focusing sound waves. 5. All radioactivity is man-made 6. Antibiotics kill viruses as well as bacteria 7a. Does the Earth go around the Sun, or does the Sun go around the Earth? 7b. How long does it take for the earth to go around the sun?
**Study 2: LSAY**
1. Antibiotics kill viruses as well as bacteria. 2. Nuclear plants destroy the ozone layer. 3. More than half of human genes are identical to those of mice. 4. Ordinary tomatoes, the ones we normally eat, do not have genes whereas genetically modified tomatoes do. 5. For the first time in recorded history, some species of plants and animals are dying out and becoming extinct. 6. Which travels faster: light or sound? 7a. Does the earth revolve around the sun, or does the sun revolve around the earth? 7b. How long does it take the earth to revolve around the sun?

#### Measuring science attitudes

Except for the GSS administered in 2006, respondents in the remaining four cohorts answered questions assessing their interest in four relevant topics: medical discoveries, scientific discoveries, space exploration, and new technologies. For each topic, participants rated their interest on a scale ranging from 1 (*not at all interested*) to 3 (*very interested*). Participants also responded to four additional items regarding more general attitudes towards science on a scale from 1(*strongly disagree*) to 4(*strongly agree*): *Because of science and technology*, *there will be more opportunities for the next generation; science makes our way of life change too fast* (reverse coded); *even if it brings no immediate benefits*, *scientific research that advances the frontiers of knowledge is necessary and should be supported by the federal government;* and *would you say that*, *on balance*, *the benefits of scientific research have outweighed the harmful results*, *or have the harmful results of scientific research been greater than its benefits*? These eight ratings were standardized and averaged, yielding a science attitudes composite (α = .69) with higher scores representing more positive attitudes towards science.

### Results

#### Relation with science knowledge

Inspection of zero-order correlations (see S1 Table) showed that religiosity was associated with lower levels of total (*r* = -.28, *p* < .001) and non-contested (*r* = -.16, *p* < .001) science knowledge and with more negative attitudes towards science (*r* = -.16, *p* < .001). Because there are a number of variables that could account for this relationship, we repeated the analysis, controlling for relevant demographic variables. As our main interest was in science knowledge excluding contested items, we regressed the science knowledge scores of the non-contested items onto religiosity while controlling for age, education, sex (0 = male, 1 = female), household income, race (two dummy coded variables), father’s education, mother’s education, region of the country (south versus all others [7]), and cohort-years (four dummy coded variables). The model was significant and accounted for about 27% of variance, *R*^2^ = .27, *F* (15, 4611) = 113.15, *p* < .001. Religiosity continued to negatively predict science knowledge while controlling for the demographic variables (see Table 2), β = -.04, *F*(1,4611) = 10.25, *p* = .001. Thus, religiosity uniquely accounts for lower levels of non-contested science knowledge even after controlling for relevant demographic variables.

**Table 2:** Multiple regression coefficients and inferential statistics for all predictors and covariates in study 1. Standardized coefficients are displayed.

Predictor	β	*t*	*p*	*Partial r*
**Age**	-.05	3.75	< .001	-.06
**Education**	.29	18.95	< .001	.27
**Father’s education**	.10	5.57	< .001	.08
**Mother’s education**	.06	3.27	< .001	.05
**Sex**	-.14	10.97	< .001	-.16
**Income**	.10	7.31	< .001	.11
**2008**	.02	1.53	.540	.02
**2010**	.02	1.16	.582	.02
**2012**	.01	.50	.449	.01
**2014**	.01	.59	.737	.01
**2016**	-.01	.59	.139	-.01
**White**	.10	5.58	< .001	.08
**Black**	-.05	2.65	.004	-.04
**Region**	-.04	3.36	.001	-.05
**Religiosity scale**	-.04	3.19	.001	-.05

#### The role of spirituality

Researchers often distinguish between religiosity and spirituality (e.g., [38]), and this distinction is relevant to the issue of science literacy. Citing unpublished research by A. Willard and A. Norenzayan, Norenzayan [39] noted that spirituality rejects religious dogma and taps into psychological experiences “…that are stripped away from the cultural baggage inherited from …traditions” (p. 479). If spirituality is less tied to religious concepts (e.g., God, scriptures, creationism) that induce opposition to science, it may not share with religion its negative relation to science knowledge. Indeed, repeating the same multiple regression analysis as above, but including an item measuring spirituality in the religiosity composite (α = .87), yielded a somewhat weaker negative relation between science knowledge and religiosity, β = -.03 (vs. β = -.04), F(1, 4611) = 3.06, *p* = .080, partial *r* = -.03.

#### Mediation analysis

**Fig 1:**
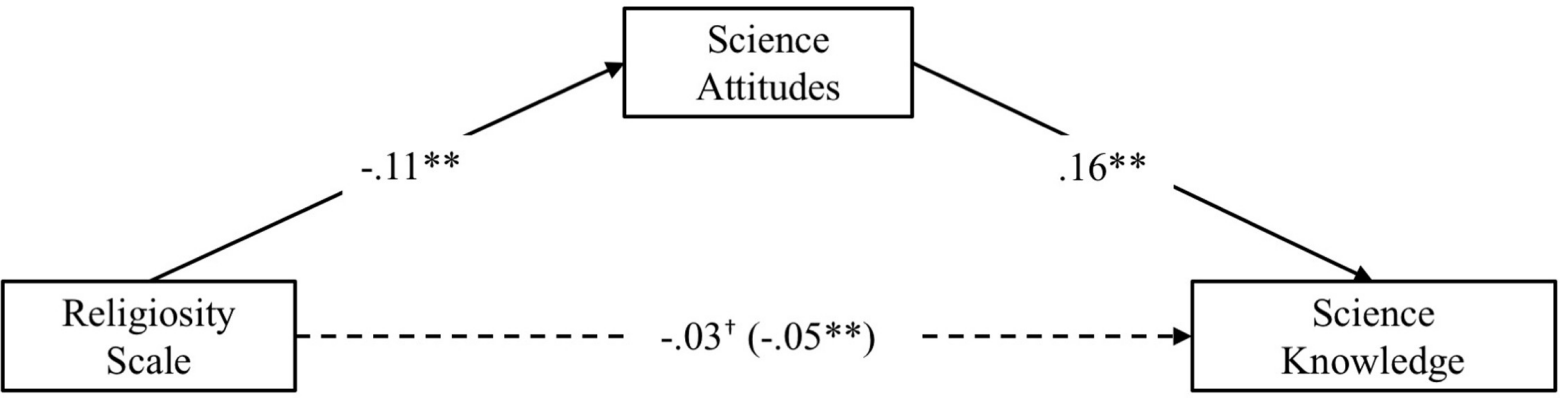
Mediation model depicting the relationship between religiosity, science attitudes, and science knowledge in stud 1. Standardized coefficients are displayed; coefficient in parentheses is the total effect before controlling for the mediator; ^†^*p* = .050, **p < .001.

To examine whether the relation between religiosity and science knowledge was accounted for by negative attitudes towards science, we conducted a mediation analysis with 5,000 bootstrapped samples, again controlling for the same demographic variables (Fig 1). Because four of the questions regarding science attitudes were not asked during the 2006 GSS, we used only the 2008–2016 cohorts in this analysis (*N* = 3,479). As shown in Fig 1, the indirect effect from religiosity to science attitudes, and then to science knowledge was significant, β = -.02, 95% CI: -.0239, -.0117, accounting for 39% of the total relation between religiosity and science knowledge; including science attitudes in the model reduced the relationship between religiosity and science knowledge from β = -.05 to β = -.03, though it remained significant. Thus, controlling for relevant demographic variables, greater religiosity was associated with lower science knowledge, a relation that was partially accounted for by less positive attitudes towards science among religious people.

It’s also important to note that, while these models are consistent with our hypothesis that religiosity is linked to lower science knowledge because of less positive attitudes towards science, cross-sectional mediation models do not indicate causation. Thus, another interpretation of this model is simply that both religiosity and science attitudes are uniquely related to science knowledge. We also tested alternative models of these relations and describe these in the supplementary file (see Figure A in S1 File).

#### The role of education

In the above mediation analysis, education served as one of the covariates. When we tested education as a mediator alongside science attitudes, the indirect effect of education was also significant (β = -.01, 95% CI: -.0162, -.0018); the indirect effect through science attitudes was still significant (β = -.02, 95% CI: -.0250, -.0130), reducing the relation between religiosity and science knowledge from β = -.05, *p* < .001 to β = -.03, *p* = .050.

### Discussion

Results from Study 1 indicate that higher religiosity is associated with lower levels of science literacy even after excluding contested items (e.g. evolution). Further, religiosity is associated with more negative attitudes towards science—an effect that partially accounts for the relation between religion and science literacy. These results are in line with the general conflict hypothesis.

Education accounted for a small portion of the relation between religiosity and science knowledge, suggesting that religiosity is associated with lower levels of science literacy resulting from less formal education. In Study 2, we further examined these relations using an independently collected dataset to replicate the effects observed here and to expand on the relation between religiosity, attitudes towards science, and science literacy.

## Study 2

The goal of study 2 was twofold. First, we examined whether a general measure of religiosity was again related to science knowledge, as well as to lower trust in scientific sources of information, and whether these relations are mediated by science attitudes. Second, we examined whether the relation between a person’s religiosity and science attitudes/knowledge (and presumably lower trust in scientific information) begins earlier, with the religiosity of that person’s parents.

### Method

#### Participants

The data were obtained from the Longitudinal Study of American Youth (LSAY [26]). This study collected two waves of data from over 3,000 American youth. The first wave, which began in 1987, collected data from two cohorts. Survey of Cohort 1 began with almost 3,000 10^th^ grade public school students and followed them for an initial period of seven years, ending about four years after high school graduation. Survey of Cohort 2 began with almost 3,000 public school students in 7^th^ grade and followed them for seven years, until about one year after high school graduation. Data collection for both cohorts ended in 1994. The second wave of data collection was carried out during 2007–2011 and included questions concerning science knowledge and attitudes towards science, as well as questions assessing religious beliefs and behaviors which were similar to the question used in the GSS.

After deleting respondents with missing data on exogenous variables, we examined responses of the 2,839 participants (1,541 females) who responded to the relevant ballot of questions used in our analyses. In 2008, participants’ ages ranged approximately from 33 to 37. Participants were 86% white, 7% African-American, and 7% Hispanic. In 2008, 3% had less than a high school education, 42% had achieved a high school education, 8% had earned a 2-year degree, 30% had earned a 4-year degree, and the remaining 17% had earned an advanced degree. A list of all the demographics controlled for in the analysis is presented in the Results section.

#### Measuring scientific knowledge

The 2008 survey included 15 science knowledge questions. Seven questions addressed the contested topics of evolution, the Big Bang, continental drift, and climate change. Accordingly, only responses to the remaining eight questions were examined in the main analysis (see bottom half of Table 1). As in Study 1, participants received one point for each correct response and zero point for incorrect or “Don’t know” responses; participants received an extra point for answering correctly question 7b (which was contingent on answering correctly question 7a). Overall scores had a 0–8 range with higher scores indicating higher science knowledge. As in Study 1, we also report the zero-order correlations between the total 15 item general science knowledge score in the supplementary materials (see S2 Table).

#### Measuring religiosity

Participants rated on 1(*strongly disagree*) to 4 (*strongly agree*) scales whether they believed that the bible is the literal word of God, and whether a personal God exists; they were also asked how often they attended religious services and activities on weekly basis. Responses to these three questions were standardized and combined into a religiosity composite (α = .69).

#### Measuring science attitudes

In the 2008 follow-up, participants completed the same measure of attitudes toward science that was administered in the GSS and used in Study 1. That is, participants rated their interest in science discoveries, science and technology, new medical discoveries, and space exploration on a scale from 1(*not at all interested*) to 3(*very interested*). In addition, participants also rated their attitudes toward science on eight more statements on a scale from 1(*strongly disagree*) to 4(*strongly agree*). Four of the items were identical to those used in Study 1: *Because of science and technology*, *there will be more opportunities for the next generation*, *science makes our way of life change too fast* (reverse coded), *even if it brings no immediate benefits*, *scientific research that advances the frontiers of knowledge is necessary and should be supported by the federal government*, *how would you assess the balance between the beneficial and harmful results of scientific research* (answered on a 5-point scale)? An additional four items were new, but similar to the previous four: *Science and technology are making our lives healthier and easier*, *most scientists want to work on things that make life better for the average person*, *would you say the world is better off or worse off because of science*?, *It is not important to know about science in everyday life* (reverse-coded).

We standardized all 12 items and combined them into a composite score representing science attitudes (α = .78) with higher scores representing more positive attitudes towards science. This is the scale that was used in subsequent analyses. However, using only the same eight items as in Study 1 produced the same results, and we discuss this analysis in the section “Revisiting Studies 1 and 2,” below.

#### Parents’ religiosity

At various times, participants’ parents (mothers and fathers) were asked four questions assessing their religious beliefs and the extent to which religion played a role in their children’s upbringing during the years 1987–1994. The questions were (1) *how many times have you attended a religious service with your child during this last year*? Parents responded with a number; (2) *how often do you talk to your child about religious values and beliefs*? Parents responded on a scale from 1(*not at all*) to 3(*often*). For those in Cohort 1, these two questions were asked during the 11th and 12^th^ grade years. For those in Cohort 2, these questions were asked each year from 7^th^ to 11^th^ grade. Parents were also asked (3) if they *believe that a personal God exists* and (4) whether they *believe the bible is the literal word of God*. They responded to both questions on a scale from 1(*strongly disagree*) to 4 (*strongly agree*). For Cohort 1, these questions were asked during the 10^th^ and 11^th^ grades, and for Cohort 2, these questions were asked during the 7^th^ and 8^th^ grades. Scores for each of these questions were standardized and averaged across the years; the four averages were then standardized and combined into a composite measuring parents’ religiosity (α = .70).

#### Measuring trust in science

During the follow-up surveys in 2009–2011, participants were asked about their trust in information from various sources. For example, participants were asked *If you wanted to get more information about the flu virus*, *how much would you trust information from each of the following sources*? The sources included news (e.g., *CNN*, *New York Times*, *NPR*), social sources (*YouTube*, *President Obama*), internet (e.g., *Wikipedia*), and scientific sources (e.g., *NASA*, *science museum*). The topic of the information changed each year and included the flu virus (2009), genetically modified organisms (GMO; 2010), the Hubble Space telescope (2011), nuclear power (2011), and climate change (2009, 2011). Participants responded on a scale ranging from 0 (*would not trust this source*) to 10 (*would definitely trust this source*). Excluding the questions about climate change, we identified 11 questions about scientific sources of information; those included information about the flu virus from a (1) science museum and (2) PBS Nova or Discovery; information about GMOs from (3) a science museum and (4) PBS Nova/Discovery; information about the Hubble from (5) Nasa, (6) a science museum, (7) PBS Nova/Discovery, and (8) an astronomy professor; information about nuclear power from (9) a science museum, (10) a science professor, and (11) PBS Nova/Discovery. Since the items were highly related, we standardized and combined them into a composite score representing trust in scientific sources of information (α = .90).

### Results

**Fig 2:**
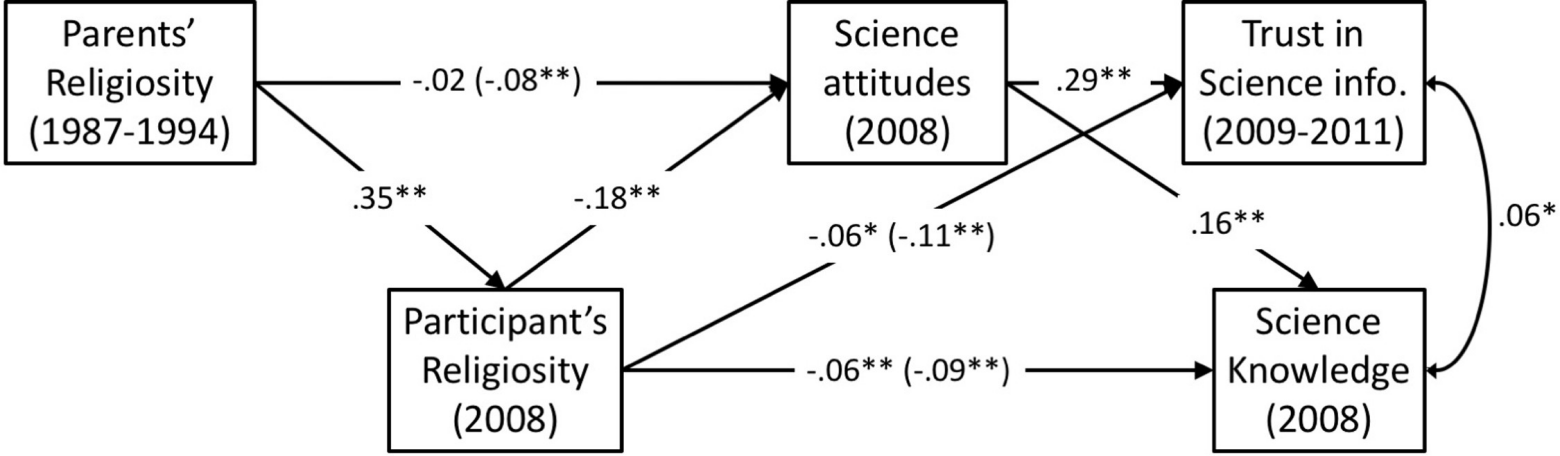
Structural equation model depicting the relationship between parents’ religiosity during high school (1997–1994) and participant’s outcomes in 2008 or later. Standardized coefficients are displayed; coefficients in parentheses are total effects, coefficients outside parentheses are the direct effects after mediators are controlled for; covariates are not displayed; **p < .001, *p < .01.

Inspection of zero-order correlations (see S2 Table) showed that religiosity was again related to more negative attitudes towards science (*r* = -.16, *p* < .001) and lower levels of both total (*r* = -.26, *p* < .001) and non-contested science knowledge (*r* = -.13, *p* < .001). Using structural equation modeling, we created a model to examine the relation between parents’ religiosity, participants’ religiosity, attitudes towards science, trust in scientific sources of information, and science knowledge. We used parents’ religiosity (as measured in 1987–1994) to predict participants’ religiosity and attitudes towards science (as measured in 2008). We also used participants’ religiosity and attitudes towards science to predict science knowledge scores and trust in scientific information. Thus, we expected to replicate the findings from Study 1, showing that the relationship between religiosity and science knowledge is mediated by attitudes towards science. Further, we examined the indirect (mediated) effects from both parents’ religiosity and participants’ religiosity to science knowledge and trust in scientific information. All analyses were conducted in MPlus v. 8 [40]. Missing data on endogenous variables were handled using full-information maximum likelihood. Fig 2 shows the model and Table 3 presents the coefficients of all the indirect effects in the model.

**Table 3:** Coefficients for all possible indirect effects from SEM model in study 2. Standardized coefficients and 95% confidence intervals are displayed.

Path	β	*p*	LLCI, ULCI
**Predicting Science Attitudes**			
Parents’ religiosity ➔ Religiosity (2008) ➔ Science attitudes	-.06	< .001	-.079, -.048
**Predicting Trust in Scientific Sources of Information**			
Religiosity (2008) ➔ Science attitudes ➔ Trust in Science info.	-.05	< .001	-.067, -.040
Parents’ religiosity ➔ Science attitudes ➔ Trust in Science Info.	-.02	< .001	-.035, -.012
Parents’ religiosity ➔ Religiosity (2008) ➔ Trust in Science Info.	-.04	< .001	-.054, -.026
Parents’ religiosity ➔ Religiosity (2008) ➔ Science attitudes ➔ Trust in Science Info.	-.02	< .001	-.061, -.027
**Predicting Science Knowledge Scores**			
Religiosity (2008) ➔ Science Attitudes ➔ Science knowledge	-.03	< .001	-.038, -.020
Parents’ religiosity ➔ Science attitudes ➔ Science Knowledge	-.01	< .001	-.019, -.006
Parents’ religiosity ➔ Religiosity (2008) ➔ Science Knowledge	-.03	< .001	-.042, -.020
Parents’ religiosity ➔ Religiosity (2008) ➔ Science attitudes ➔ Science Knowledge	-.01	< .001	-.047, -.022

We conducted the analysis using 5,000 bootstrapped samples (non-bias corrected). To control for demographic variables, we regressed each of our endogenous variables onto region (north vs. south), gender, race (two dummy codes for Hispanic and black), education (as of 2008), and parents’ education levels; these effects are not depicted in the figure. Using standard fit criteria [41], the model fit the data well, χ^2^ (4) = 12.10, *p* = .017, RMSEA = .027, CFI = .996, SRMR = .007. Similar to Study 1, the model explained about 29% of variance in science knowledge (*R*^2^ = .29, *p* < .001). At the request of a reviewer, a model controlling also for political orientation (see S1 File) and removing the literal belief items (see ‘Revisiting Studies 1 and 2’, below) was also tested; it showed highly similar relations to those reported here.

As shown in Fig 2 and Table 3, this model provides a replication of the effects reported in Study 1. Participants’ religiosity (in 2008) predicted lower science knowledge scores, β = -.09, *p* < .001, and the indirect effect to science knowledge via science attitudes was significant (see Table 3). Including science attitudes as a mediator accounted for about 54% of the total effect and reduced the relationship between religiosity and science knowledge from β = -.09 to β = -.06, though it remained significant, β = -.06, *p* < .001. We also conducted additional analyses to directly replicate the mediation models using only the common items between the GSS and LSAY datasets. These analyses are reported in the section ‘Revisiting Studies 1 and 2’, below.

Next, religiosity (in 2008) also predicted having less trust in scientific sources of information, β = -.11, *p* < .001. Positive attitudes toward science predicted more trust in scientific sources of information, β = .29, *p* < .001, and the indirect effect via science attitudes was significant (see Table 3). Including science attitudes as a mediator accounted for about 54% of the total effect, reducing the relationship between religiosity and trust in scientific sources of information from β = -.11 to β = -.06, though it remained significant, β = -.06, *p* = .002.

Parents’ religiosity (as reported by the parents in 1987–1994) predicted participant’s negative attitudes towards science (in 2008), β = -.08, *p* < .001, and the indirect effect via the participant’s religiosity (also in 2008) was significant. Including participant’s religiosity as a mediator between parents’ religiosity and science attitudes explained 75% of this effect, reducing the relationship to non-significance, β = -.02, *p* = .399.

Finally, the indirect relation from parents’ religiosity to science knowledge was significant through all three routes: via participants’ religiosity (in 2008), via science attitudes (in 2008), and through participants’ religiosity to science attitudes, and then to science knowledge. Similarly, the same three indirect paths from parent’s religiosity to trust in scientific information were also significant (see Table 3).

#### The role of education

In the analyses above, education served as one of the covariates. When we tested education as a mediator alongside science attitudes in a simple mediation model (including all covariates), the indirect effect of education was not significant (β = -.01, 95% CI: -.0243, .0039); the indirect effect through science attitudes remained significant (β = -.04, 95% CI: -.0509, -.0290), reducing the relation between religiosity and science knowledge from β = -.13, *p* < .001, to β = -.09, *p* < .001.

### Discussion

These results both replicate and extend the effects found in Study 1. Specifically, religiosity was again associated with lower levels of both contested and non-contested science knowledge, with more negative attitudes towards science, and with less trust in scientific sources of information. These results suggest that religiosity is associated with a less positive view of science, which results in less science learning. These effects were obtained for non-contested science topics, lending additional support to the general conflict hypothesis.

In addition to replicating the effects from Study 1, we also explored additional longitudinal relations between religiosity and science attitudes and knowledge. We found that negative attitudes towards science may begin early in life, with the religious beliefs of one’s parents. That is, the data here suggests that growing up in a religious household may lead to less positive views of science, resulting in lower levels of science knowledge and less trust in scientific information. This sequence is consistent with a directional model in which religiosity leads to a more negative view of science and to less science knowledge.

In the analyses that follow, we first examined a direct replication between the data from Studies 1 and 2, using only the common items between the studies.

## Studies 1 and 2 revisited

The religiosity composites in studies 1 and 2 included two religiosity items that were common across the two data sets. In both surveys, participants were asked if they believed in a personal god, and how often they attend religious services. As these two items represent central themes in measures of religiosity, it was of interest to test the relation between a religiosity composite made of these two items and non-contested science knowledge. In addition to items regarding belief in God and attendance of religious services, Study 2 included a third item concerning participants’ belief in the bible (i.e., *The bible is the actual word of God and is to be taken literally word for word*). As literal interpretation of the bible is a feature of fundamentalism [7, 42], it might be argued that the results are partly due to fundamentalism. Therefore, it is important to examine whether the data show a negative relation between religiosity and science knowledge, using a religiosity measure without the biblical literalism item.

Both datasets included the same four-item measure of science interest, and almost the same four items measuring attitudes towards general science. We, therefore used these common items to construct the science attitudes composite. Finally, the science knowledge measure was identical across the two studies.

For both the GSS and the LSAY data, we standardized and then combined responses to the questions about belief in God and attendance of religious services (αs = .64 and .46, respectively). We also standardized and then combined responses to the eight items that formed the science attitudes scale (for GSS: α = .69; for LSAY: α = .74). Using these measures of religiosity and science interest as well as our previous measure of science knowledge, we repeated the mediation analyses with both the GSS and the LSAY data, separately. We controlled for the same demographic variables as reported in the previous analyses.

**Fig 3:**
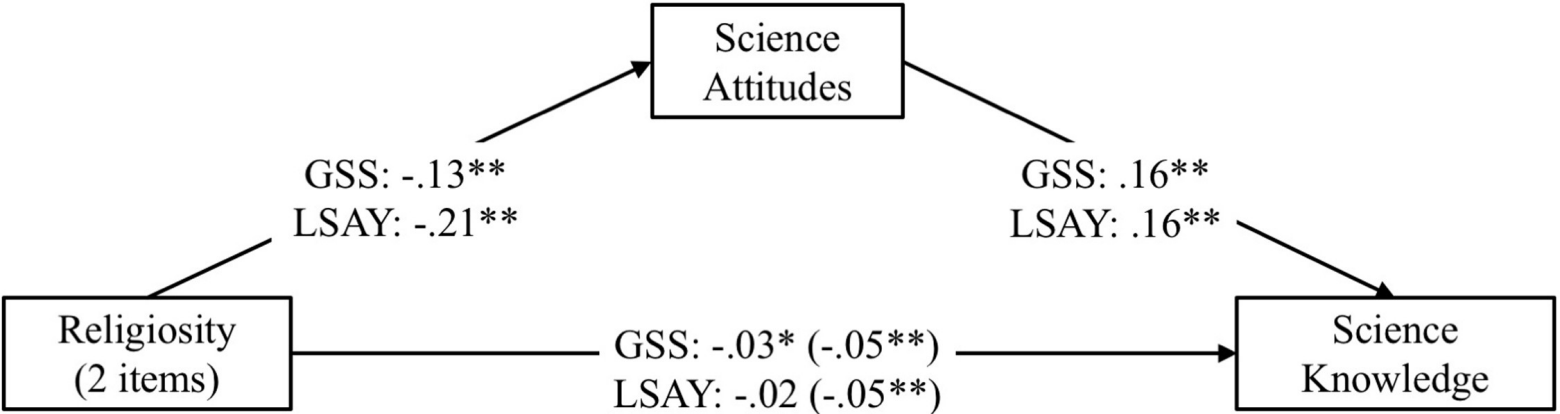
Replications of the mediation models depicting the relationship between religiosity, science attitudes, and science knowledge for studies 1 and 2. Standardized beta coefficients are displayed; coefficients in parentheses are the total effects before the mediator is controlled for; **p < .001.

The results show significant negative relations between religiosity and science knowledge in both the GSS and the LSAY data (see Fig 3). The results also show significant indirect effects through science attitudes for both studies. For the GSS: β = -.02, 95% CI: -.0276, -.0141; for the LSAY: β = -.03, 95% CI: -.0448, -.0244; the indirect effects through science attitudes explain 40% and 31% of the total effects in the two studies, respectively. Controlling for the mediator, the direct relation between religiosity and science knowledge was reduced from β = -.05 to β = -.03 (Study 1) and from β = -.05 to β = -.02 (Study 2). As with Study 1, we also tested alternative models where science knowledge was treated as the mediator (see Figure A in S1 File.).

### Discussion

These results replicate our previous findings for both Study 1 and Study 2 and demonstrate that the observed relations do not depend on fundamentalist or literalist forms of belief. Next, we turn to replication of these results in different samples and using another measure of religiosity, but one that also focuses on beliefs and practice.

## Study 3

The purpose of Study 3 is to replicate the effects observed in Studies 1 and 2 using another independent dataset and another measure of religiosity; this measure is widely used [23] and does not include items about fundamentalist or literalist beliefs.

Data for Study 3 were collected for a larger survey on general aspects of science rejection with a focus on beliefs about genetically modified foods. Other aspects of this survey will be reported elsewhere [43] but the relations described here are not described elsewhere. The surveys also included the relevant measures of science knowledge questions, religiosity, and demographic controls.

### Participants

Participants (*n* = 747) were recruited simultaneously from the undergraduate subject pool at a northeastern university (*n =* 262) and from the Research Match online recruitment system (*n* = 485). There were 178 males and 532 females (4 identified as other). Participants ranged in age from 18–93 (*M* = 41.30, *SD* = 19.85) and were 76% white, 13% Asian, 3% Black, 3% Hispanic, and 4% mixed or other. Eight percent reported having a high school education, 32% some college, 5% a two-year degree, 19% a 4-year degree, 8% some post-graduate education, and 28% had an advanced degree.

### Materials and procedure

Participants were recruited for a survey regarding beliefs about science where they filled out questionnaires regarding their knowledge and beliefs about genetically modified foods. All questionnaires were presented in randomized order, except that demographic questions were presented last. Among these questionnaires was the 18-item measure of science knowledge, composed of the 13 non-contested items, as well as the 5 contested items, common between Studies 1 and 2. The items were summed up to create a general science knowledge score ranging from 0 to 18, as well as a non-contested science knowledge score ranging from 0–13. Other measures are not relevant to the current study and are not discussed further.

Demographics questions included a 6-item measure of religiosity (e.g., *I believe in God*; α = .96; [23]). Participants also answered questions about their socioeconomic standing during childhood and currently, their political orientation regarding social issues, economic issues, and voting history (α = .90), and their parents’ education levels (α = .79). Throughout the survey, there were 6 attention check questions (e.g., *select ‘somewhat agree’ and continue on;* [44]); excluding those who missed at least one attention check question did not change the results. Both sets of results are reported below.

### Results and discussion

Replicating the results from Studies 1 and 2, religiosity correlated negatively with general science knowledge (*r* = -.30, *p* < .001) and with non-contested science knowledge (*r* = -.20, *p* < .001). We then regressed the non-contested science knowledge onto religiosity while controlling for data source, age, education, gender (two dummy codes), parents’ education, current and childhood SES, political orientation, and race (4 dummy codes).

The model was significant and explained about 21% of variance in non-contested science knowledge, *R*^2^ = .21, *F* (14, 694) = 12.78, *p* < .001. Religiosity continued to uniquely predict lower levels of non-contested science knowledge. Coefficients are displayed in Table 4, below. An additional analysis with the same controls but excluding those who missed at least one attention check item (*n* = 32), also showed a significant relation between religiosity and non-contested science knowledge: β = -.13, *F* (1, 662) = 10.11, *p* = .002, *partial r =* -.12. These results replicate the negative relation between religiosity and science literacy, using a somewhat different measure of religiosity.

**Table 4:** Multiple regression coefficients indicating the negative relation between religiosity and science knowledge in study 3. Standardized coefficients are displayed.

Predictor	β	*t*	*p*	*partial r*
**Source**	.11	1.60	.110	.06
**Age**	-.06	-1.04	.300	-.04
**Education**	.21	4.23	< .001	.16
**Male**	-.10	-0.55	.586	-.02
**Female**	-.34	-1.87	.062	-.07
**Parents’ education**	.10	2.48	.013	.09
**Current SES**	.08	2.08	.038	.08
**Childhood SES**	.05	1.47	.142	.06
**White**	.11	1.56	.120	.06
**Black**	-.10	-2.36	.018	-.09
**Hispanic**	-.08	-1.91	.057	-.07
**Asian**	-.07	-1.02	.310	-.04
**Religiosity**	-.19	-5.19	< .001	-.19

#### The role of education

In the analyses above, education served as one of the covariates. When we tested education as a mediator in a simple mediation model (including all covariates), the indirect effect of education was significant (β = -.01, 95% CI: .0034, .0339), reducing the relation between religiosity and science knowledge from β = -.15, *p* < .001, to β = -.16, *p* < .001.

## Study 4

Data for Study 4 were collected as part of a larger study for scale development and is part of a currently ongoing research program [45]. This survey also included the relevant measures of religiosity and science knowledge, as well as the same measure of science attitudes used in Study 1. The present data have not been described or reported elsewhere.

### Participants

Participants (*n* = 992) were recruited simultaneously from the undergraduate subject pool at a northeastern university (*n =* 375) and from the Research Match online recruitment system (*n* = 617). There were 231 males and 723 females (11 identified as other). Participants ranged in age from 18–86 (*M* = 38.78, *SD* = 19.21) and were 72% white, 13% Asian, 4% Black, 4% Hispanic, and 4% mixed or other. Ten percent reported having a high school education, 33% some college, 6% a two-year degree, 19% a 4-year degree, 6% some post-graduate education, and 25% had an advanced degree.

### Materials and procedures

The survey collected information on general beliefs about science topics, such as science attitudes and beliefs about climate change, vaccinations, alternative medicine, etc. Again, only the items relevant to the current study are described here. Participants completed the same 18-item measure of science knowledge from Study 3; this was scored into an 18-item general science knowledge score and a 13-item non-contested science knowledge score. Participants also responded to a shortened (6-item) version of the science attitudes measure used in the previous studies (α = .66). The six items were: *Scientific research makes life change too fast*, *the benefits of scientific research outweigh any possible harms*, *the world is better because of science*, *science and technology make more opportunities for the next generation*, *scientists want to make life better*, and *it is not important to know about science in daily life*.

Participants also responded to the same demographic controls as in Study 3: childhood and current SES, the three-item measure of political orientation (α = .89), parents’ education levels (α = .80), and the 6-item religiosity scale used in Study 3 (α = .96; [23]). Finally, 11 attention check questions were included as in Study 3 [44], so those who missed at least one item will be excluded. Results with and without exclusion criteria were similar and both are reported below.

### Results

#### Main hypothesis

Replicating the results from studies 1–3, religiosity negatively correlated with general (*r* = -.30, *p* = < .001) and non-contested (*r* = -.18, *p* < .001) science knowledge, and negatively with science attitudes (*r* = -.12, *p* < .001). As before, we regressed the non-contested science knowledge score onto religiosity while controlling for data source, age, education, gender (two dummy codes), parents’ education, current and childhood SES, political orientation, and race (4 dummy codes). The model was significant and explained about 19% of variance in non-contested science knowledge, *R*^2^ = .19, *F* (14, 943) = 15.68, *p* < .001. Religiosity continued to uniquely predict lower levels of non-contested science knowledge. Coefficients are displayed in Table 5, below. In an additional analysis with the same controls, excluding those who missed at least one attention check item (*n* = 32) also showed a significant negative relation between religiosity and non-contested science knowledge: β = -.13, *F* (1, 859) = 13.73, *p* < .001, *partial r =* -.13.

**Table 5:** Multiple regression coefficients indicating the negative relation between religiosity and science knowledge in study 4. Standardized coefficients are displayed.

Predictor	β	*t*	*p*	*partial r*
**Source**	-.05	-0.83	.409	-.03
**Age**	-.09	-1.79	.074	-.06
**Education**	.30	7.01	< .001	.22
**Male**	.02	0.13	.898	.00
**Female**	-.18	-1.42	.157	-.05
**Parents’ education**	.10	2.76	.006	.09
**Current SES**	.09	2.74	.006	.09
**Childhood SES**	-.05	-1.41	.159	-.05
**White**	.01	0.18	.859	.01
**Black**	-.09	-2.16	.031	-.07
**Hispanic**	-.07	-1.64	.102	-.05
**Asian**	-.13	-2.27	.023	-.07
**Religiosity**	-.16	-5.24	< .001	-.17

#### Mediation analysis

**Fig 4:**
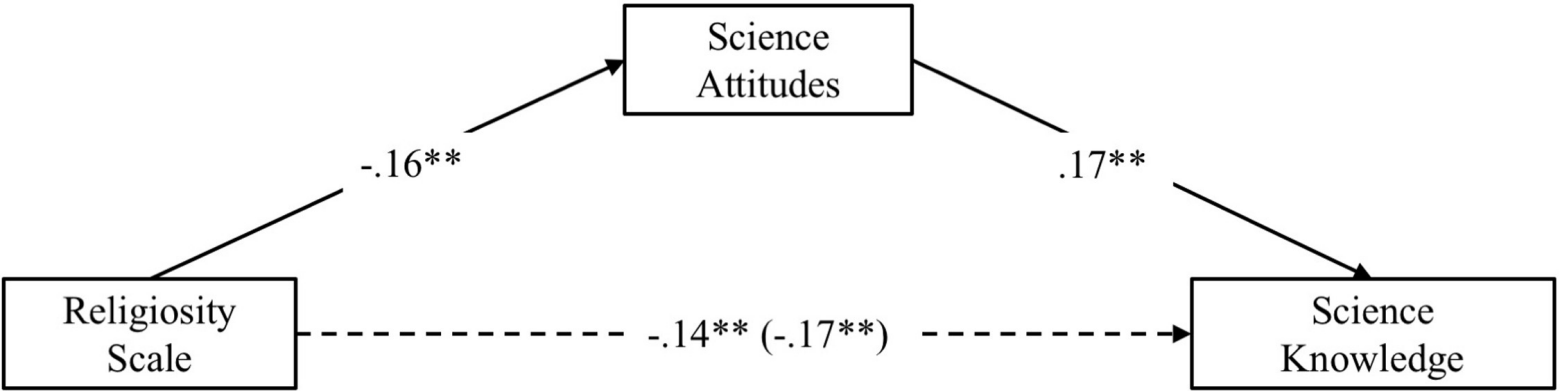
Mediation analysis depicting the relations between religiosity, science attitudes, and non-contested science knowledge in study 4. Standardized coefficients are displayed; **p < .001.

As in Studies 1 and 2, I conducted a mediation analyses using the religiosity scale as the independent variable, science attitudes as the mediator, and non-contested science knowledge as the dependent variables. We again controlled for data source, age, education, gender, race, parents’ education, current and childhood SES. As shown in Fig 4, below, the indirect effect through science attitudes was significant, β **=** -.03, 95% CI: -.0440, -.0134. Controlling for science attitudes reduced the relation between religiosity and science knowledge from β **=** -.17 to β **=** -.14, and accounted for about 18% of the relation between religiosity and non-contested science knowledge. As with Studies 1 and 2, we also tested an alternative model where science knowledge was the mediator between science attitudes and science knowledge (see Figure A in S1 File).

#### The role of education

In the analyses above, education served as one of the covariates. When we tested education as a mediator alongside science attitudes in a simple mediation model (including all covariates), the indirect effect of education was not significant (β < .01, 95% CI: -.0144, .0240); the indirect effect through science attitudes remained significant (β = -.02, 95% CI: -.0326, -.0017), reducing the relation between religiosity and science knowledge from β = -.16, *p* < .001, to β = -.15, *p* < .001.

#### Discussion

These results provide a direct replication of the negative relation between religiosity and science knowledge observed in Study 3, and a conceptual replication of the results observed in Studies 1–2. This relation was larger in Studies 3 and 4 than in studies 1 and 2, perhaps because of differences between the samples and or the religiosity measure that was used.

Though the science attitudes scale was not available in Study 3, it was included in Study 4. Results suggest that a negative relation between religiosity and science attitudes partially accounts for the negative relation between religiosity and science knowledge; this indirect effect is similar in magnitude to the indirect effects observed in Studies 1 and 2.

## General discussion

The findings from these four studies show that religiosity is negatively related to science knowledge and is associated with more negative attitudes towards science. Importantly, these results were obtained while controlling for a large number of demographic variables, and after deleting contested portions of science knowledge. All four studies are correlational. However, the relation of parents’ reports of their religiosity and the religious upbringing of their children with (some 20 years later) their children’s attitudes toward science (Study 2) implies that religiosity may impact attitudes towards science, and thus science knowledge, later in life.

We measured religiosity differently across the four studies. The religiosity measures in studies 1 and 2 were identical as were the measures used in studies 3 and 4. Previous researchers examined the relation between religiosity and science knowledge, measuring religiosity via specific combinations of religious affiliation [10] and fundamental/literalist interpretations [5, 9]. The present results demonstrate that the negative relation between religiosity is general and robust across different samples and broad religiosity measures that focus on belief in God and religious practices (attendance, prayer)—quintessential elements of religiosity [21–24].

Of course, the differences between the present study and past research may also be understood in light of differences between how sociologists and social psychologists approach research. Whereas sociologists may be more interested in the social structure of religious traditions or affiliations, we have approached this question with a focus on individual variation in the strength of religious belief and practice. These are two different ways of asking the same question which both shed light on the relation between religion and science. Echoing other researchers [6,7], we hope for more research in psychology and in other disciplines on this issue, both in and outside of the US.

Our findings indicate that one possible mediator of the negative relation between religiosity and science knowledge is negative attitudes towards science. Our interpretation of this relation is straightforward: to the extent that science is viewed as less interesting, useful, or valid, one would be less likely to learn about it. Longitudinal findings from Study 2 suggests these negative attitudes towards science may begin early in life when one is exposed to their parents’ religious beliefs. Future research will be needed to examine exactly how religious upbringing may negatively impact science attitudes.

However, it’s important to note that cross-sectional mediation models do not indicate causation because we did not manipulate religiosity or science attitudes in these studies. In line with this consideration, we presented in the supplementary file formal tests of alternative models. Some resolution of this issue may be obtained only with longitudinal research.

In each study, we also tested whether the relation between religiosity and science knowledge could be accounted for by formal education level. However, education level did not consistently account for additional variance above science attitudes. While some previous research suggests that religion may lead one to pursue less education [36], the present findings are not necessarily in conflict with such reports. In each of the four studies, religiosity was negatively correlated with education but the relation between education and science knowledge was weaker and inconsistent. This is likely because not all education focuses on science such that education in the humanities and arts, for example, may not contribute to science knowledge. However, much more research will be needed to disentangle the roles of education, science education, and influences of religion on interest in science, which likely would be related to whether one pursues science education. Another limitation to consider is that all of the studies described herein are based in the US. Future research will need to consider country-level and cultural differences as moderators of the relation between religiosity and science knowledge.

Thus, the picture that emerges is that religiosity is associated with less interest in science and the believe that science is less important; such attitudes are related to somewhat lower levels of science literacy and less trust in scientific sources of information. Following Evans [5], this finding suggests that, as religion offers its own route to knowledge, people of faith might be less interested in what science has to offer. However, because the mediation was partial, it is clear there are other routes leading from religiosity to lower science knowledge. For example, one study [46] found that threatening stereotypes about Christians’ low competence in science may lead them to underperform in science. Elsdon-Baker [47] suggested that the proliferation of such stereotypes may actually “create creationists” by reinforcing the belief that Christians don’t belong in science, thus promoting identification with counter-scientific beliefs. Sherkat [42] suggested that tight religious communities might actually prevent access to science knowledge because they deem it untrustworthy.

In view of the present findings, the challenge is how to increase science knowledge in the face of religious disinterest or actual opposition. Improving science knowledge and literacy will allow people to make more informed, evidence-based decisions about choices, beliefs, and activities, as well as about the products and services they use or avoid. According to the present result, the recent decline in religiosity [48] might help but, given that the majority of the world population still defines itself as religious, it behooves us to find a way for people to keep their religious beliefs and yet open their minds to science.

## Supporting information

S1 Table Correlations between all variables in Study 1. **p < .001, *p < .05, ^†^*p* < .10.
(PDF)

S2 Table Correlations between all variables in Study 2. **p < .001, *p < .05, ^†^*p* < .10.
(PDF)

S1 File Supplementary analyses document. (DOCX)
