# Supplementary material for: Religiosity predicts negative attitudes towards science and lower levels of science literacy
Source: PLoS One. 2018 Nov 27;13(11):e0207125. doi: 10.1371/journal.pone.0207125 (PMC6258506; doi:10.1371/journal.pone.0207125)
Supplement: S1 Table — **p < .001, *p < .05, †p < .10. (PDF) [file pone.0207125.s001.pdf]

S1 Table. Correlations between all variables in Study 1. **p < .001, **p < .05, †p < .10.

	1	2	3	4	5	6	7	8	9	10	11	12	13	14	15	16	17	18
**1. Religiosity**																		
**2. Science attitudes**	-.158**																	
**3. Non-contested science knowledge**	-.160**	.299**																
**4. Total science knowledge**	-.284**	.346**	.924**															
**5. Age**	.178**	-.032*	-.103**	-.115**														
**6. Education**	-.093**	.270**	.413**	.422**	-.045**													
**7. Father's education**	-.160**	.215**	.333**	.348**	-.279**	.442**												
**8. Mother's education**	-.142**	.200**	.312**	.318**	-.273**	.415**	.685**											
**9. Sex**	.164**	-.073**	-.159**	-.176**	.009	.017	-.019	-.036*										
**10. Income**	-.040**	.137**	.280**	.270**	.008	.379**	.218**	.208**	-.065**									
**11. 2006**	.036*	.065**	.000	.019	-.029†	.026	.013	.005	.000	-.021								
**12. 2008**	.019	-.016	0.01	.018	-.046**	-.042**	-.029*	-.022	-.029*	.005	-.246**							
**13. 2010**	-.026†	-.001	.018	.044**	-.023	.011	-.006	-.005	.006	-.024	-.211**	-.157**						
**14. 2012**	-.004	-.021	.002	-.033*	.021	-.006	-.002	-.006	-.023	-.003	-.222**	-.165**	-.141**					
**15. 2014**	-.009	-.044**	-.012	.027	.034*	-0.01	-.001	-.001	.009	.004	-.257**	-.191**	-.164**	-.172**				
**16. 2016**	-.027†	-.032*	-.021	-.037*	.062**	.004	.016	.02	.034*	.034*	-.421**	-.313**	-.268**	-.283**	.610**			
**17. White**	-.112**	.075**	.205**	.205**	.139**	.094**	.126**	.145**	-.019	.159**	.007	.009	.028†	-.005	-.013	-.028		
**18. Black**	.163**	-.063**	-.181**	-.198**	-.044**	-.067**	-.074**	-.039**	.027†	-.151**	.001	.000	-.015	-.004	-.011	.012	-.695**	
**19. Region**	.220**	-.046**	-.120**	-.155**	.008	-.080**	-.094**	-.072**	.02	-.069**	-.022	.034*	.012	-.01	.003	-.007	-.110**	.198**
