# Supplementary material for: Religiosity predicts negative attitudes towards science and lower levels of science literacy
Source: PLoS One. 2018 Nov 27;13(11):e0207125. doi: 10.1371/journal.pone.0207125 (PMC6258506; doi:10.1371/journal.pone.0207125)
Supplement: S2 Table — **p < .001, *p < .05, †p < .10. (PDF) [file pone.0207125.s002.pdf]

S2 Table. Correlations between all variables in Study 2. **p < .001, **p < .05, †p < .10.

	1	2	3	4	5	6	7	8	9	10	11	12
**1. Religiosity (2008)**												
**2. Parent religiosity**	.290**											
**3. Science attitudes**	-.155**	-.085**										
**4. Non-contested science knowledge**	-.132**	-.053**	.277**									
**5. Total science knowledge**	-.258**	-.155**	.366**	.873**								
**6. Education**	-.080**	.002	.186**	.411**	.420**							
**7. Father's education**	-.096**	-.055**	.164**	.258**	.276**	.388**						
**8. Mother's education**	-.085**	-.047**	.143**	.260**	.282**	.371**	.480**					
**9. Sex**	-.099**	-.029*	.124**	.210**	.214**	-.026	.006	.035**				
**10. Hispanic**	.011	.013	-.059**	-.081**	-.071**	-.096**	-.045**	-.072**	.038**			
**11. Black**	.140**	.149**	-.084**	-.224**	-.226**	-.112**	-.065**	-.082**	-.031*	-.117**		
**12. Other race**	-.114**	-.126**	.106**	.228**	.222**	.154**	.083**	.116**	-.003	-.625**	-.702**	
**13. Region**	.115**	.169**	.031†	-.058**	-.086**	-.054**	-.005	-.038**	-.006	.008	.248**	-.201**
