# Supplementary material for: Religiosity predicts negative attitudes towards science and lower levels of science literacy
Source: PLoS One. 2018 Nov 27;13(11):e0207125. doi: 10.1371/journal.pone.0207125 (PMC6258506; doi:10.1371/journal.pone.0207125)
Supplement: S1 File — (DOCX) [file pone.0207125.s003.docx]

**Supplementary Analysis File**

We also tested alternative mediation models for studies 1, 2, and 4. It’s possible that lower levels of science knowledge may lead people to be less interested in science. While cross-sectional mediation is not an indicator of causality and should not be interpreted as such, we present these models to offer a clearer description of the relation between the variables at hand.

We conducted the same PROCESS model as described in the main text, except we reversed the positions of science knowledge and science attitudes. For Studies 1 and 2, we used the 2-item religiosity composite so that the scores are directly comparable. For Study 4, we used the 6-item religiosity scale employed in that study. In each model, we controlled for relevant demographics as described in the main text.

As can be seen in Figure A, below, science knowledge accounted for about 8%, 4%, and 19% of variance in science attitudes in Studies 1, 2, and 4, respectively. In contrast, science attitudes accounted for 40%, 31%, and 18% of variance in science knowledge, suggesting it is a better predictor of science knowledge compared to the other way around.

Again, the mediation models here do not provide evidence of causality because we did not manipulate religiosity. However, they are consistent with our hypothesized model where religious people know less about science *because* they are less interested in science. Another interpretation of these models is simply that both science attitudes and religiosity are unique predictors of science knowledge because both remain significant while controlling for the other in a regression equation.

**Fig A. Mediation models depicting alternative paths between religiosity, science attitudes, and science knowledge.** ***p* < .001 , **p* < .05.

**
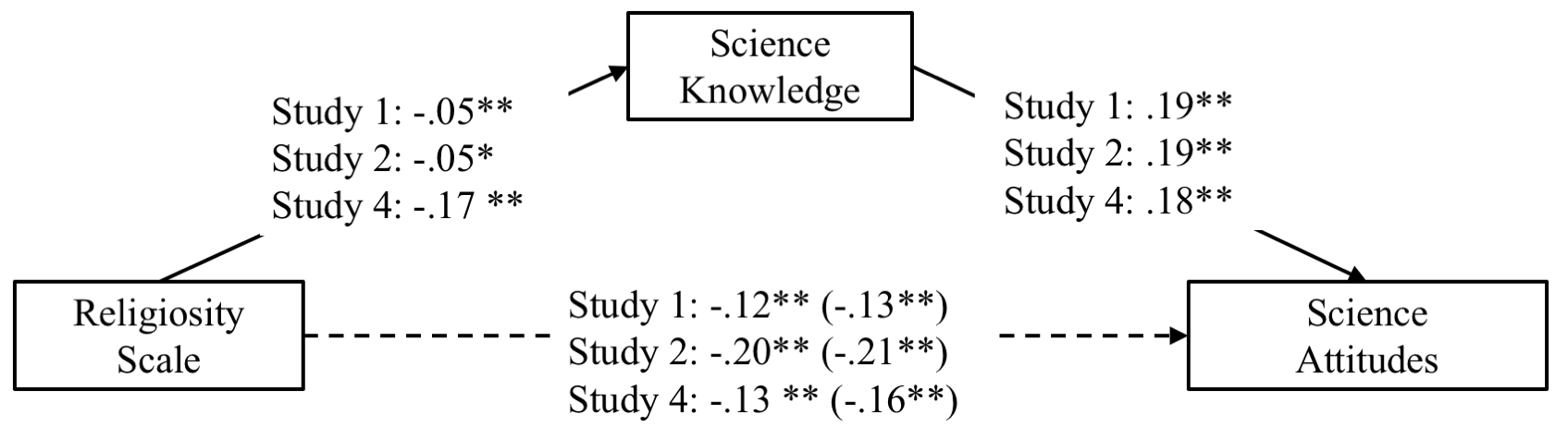
**

At the request of a reviewer, I conducted an additional analysis in which I removed the literal belief items from parents’ and participants’ religiosity scores. It should be noted that this analysis is already reported in the main text (see “Revisiting Studies 1 and 2”). It was also requested separately that I control for political orientation in the trust in scientific sources of information. It should also be noted that the trust in scientific sources of information variable does not contain any political sources nor does it contain any politically oriented topics. Nonetheless, I present the modified model below with these three accommodations.

The model is that same as that reported in the main text except I also regressed political orientation onto the two religiosity variables and trust in scientific sources of information. Further, parent’s religiosity does not include the literal belief item (3-item α = .67) and participants’ religiosity does not include the literal beliefs item (2-item α = .46). The data fit this model well, χ^2^ (6) = 53.64, *p* < .001, RMSEA = .057, CFI = .978, SRMR = .018. Similar to Study 1, the model explained about 24% of variance in science knowledge (*R*^2^ = .24, *p* < .001). The coefficients do not differ meaningfully from those reported in the main text.

**Fig B.** **SEM model excluding literal belief items and controlling for political orientation.** Standardized coefficients are displayed; ****p* < .001, ***p* < .01 , **p* < .05.


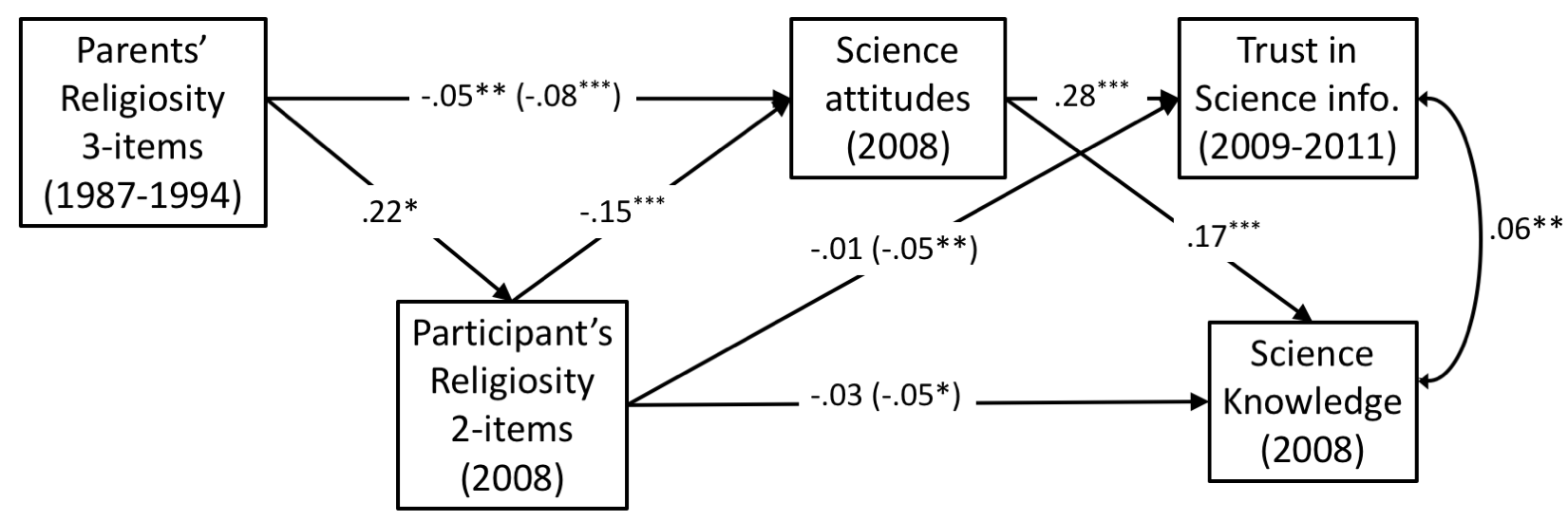


**Table A.** **All possible indirect effects in the SEM model excluding literalist belief items and controlling for political orientation.**

| **Path** | **β** | ***p*** | **[LLCI, ULCI]** |
| --- | --- | --- | --- |
| **Predicting Science Attitudes** | | | |
| Parents’ religiosity 🡪 Religiosity (2008) 🡪 Science attitudes | -.03 | < .001 | [-.049, -.023] |
| **Predicting Trust in Scientific Sources of Information** | | | |
| Religiosity (2008) 🡪 Science attitudes 🡪 Trust in Science info. | -.04 | < .001 | [-.058, -.030] |
| Parents’ religiosity 🡪 Science attitudes 🡪 Trust in Science Info. | -.02 | < .001 | [-.035, -.011] |
| Parents’ religiosity 🡪 Religiosity (2008) 🡪 Trust in Science Info. | -.01 | .033 | [-.021, -.001] |
| Parents’ religiosity 🡪 Religiosity (2008) 🡪 Science attitudes 🡪 Trust in Science Info. | -.01 | .001 | [-.013, -.006] |
| **Predicting Science Knowledge Scores** | | | |
| Religiosity (2008) 🡪 Science Attitudes 🡪 Science knowledge | -.03 | < .001 | [-.036, -.017] |
| Parents’ religiosity 🡪 Science attitudes 🡪 Science Knowledge | -.01 | < .001 | [-.021, -.006] |
| Parents’ religiosity 🡪 Religiosity (2008) 🡪 Science Knowledge | -.01 | .003 | [-.020, -.004] |
| Parents’ religiosity 🡪 Religiosity (2008) 🡪 Science attitudes 🡪 Science Knowledge | -.01 | < .001 | [-.008, -.004] |
